# Grafting Cucumber Onto Pumpkin Induced Early Stomatal Closure by Increasing ABA Sensitivity Under Salinity Conditions

**DOI:** 10.3389/fpls.2019.01290

**Published:** 2019-11-11

**Authors:** Mengliang Niu, Shitao Sun, Muhammad Azher Nawaz, Jingyu Sun, Haishun Cao, Junyang Lu, Yuan Huang, Zhilong Bie

**Affiliations:** ^1^Key Laboratory of Horticultural Plant Biology, Ministry of Education/College of Horticulture and Forestry Sciences, Huazhong Agricultural University, Wuhan, China; ^2^Department of Horticulture, College of Agriculture, University of Sargodha, Sargodha, Pakistan

**Keywords:** salt tolerance, abscisic acid, stomata, grafting, cucumber, rootstock

## Abstract

During early periods of salt stress, reduced stomatal opening can prevent water loss and wilting. Abscisic acid (ABA) signal plays an important role in this process. Here, we show that cucumber grafted onto pumpkin exhibits rapid stomatal closure, which helps plants to adapt to osmotic stress caused by salinity. Increased ABA contents in the roots, xylem sap, and leaves were evaluated in two grafting combinations (self-grafted cucumber and cucumber grafted onto pumpkin rootstock). The expression levels of ABA biosynthetic or signaling related genes *NCED2* (9-cis-epoxycarotenoid dioxygenase gene 2), *ABCG22* (ATP-binding cassette transporter genes 22), *PP2C* (type-2C protein phosphatases), and *SnRK2.1* (sucrose non-fermenting 1-related protein kinases 2) were investigated. Results showed that a root-sourced ABA signal led to decreased stomatal opening and transpiration in the plants grafted onto pumpkin. Furthermore, plants grafted onto pumpkin had increased sensitivity to ABA, compared with self-grafted cucumbers. The inhibition of ABA biosynthesis with fluridon in roots increased the transpiration rate (Tr) and stomatal conductance (Gs) in the leaves. Our study demonstrated that the roots of pumpkin increases the sensitivity of the scion to ABA delivered from the roots to the shoots, and enhances osmotic tolerance under NaCl stress. Such a mechanism can be greatly exploited to benefit vegetable production particularly in semiarid saline regions.

## Introduction

Salinity is one of the major limitations of crop production worldwide. More than 800 million hectares of agricultural land suffer from soil salinity (Rengasamy, 2010). Plant growth responds to salt stress in two phases, namely, the osmotic phase that inhibits water uptake by roots due to osmotic pressure in soil and subsequent ionic phase, during which the level of accumulated toxic ions in plants exceeds the threshold level and leads to ion toxicity or ion imbalance (Munns and Tester, 2008).

Sodium is one of the most common toxic ions existing in soil. Although several membrane transporters involved in Na^+^ uptake and transport in plants are well studied, most salinity-related studies are focused on investigating the ionic phase and discussing the control of Na^+^ transport (Fraile-Escanciano et al., 2010; Oh et al., 2010), K^+^/Na^+^ balance (Venema et al., 2002; Yue et al., 2012), and Na^+^ compartmentation (James et al., 2006; Bassil et al., 2011; Wu et al., 2019).

Several studies found that stomata play an important role in salt stress tolerance (Chen and Gallie, 2004; Rajendran et al., 2009; Rahnama et al., 2010). Stomata regulate transpirational water loss in plants (Wilkinson and Davies, 2002) and control carbon dioxide assimilation rate (Duan et al., 2015). Thus, stomatal conductance is a reliable indicator for evaluating plant salt tolerance. Using this indicator, Rahnama et al. (2010) found a positive correlation between relative stomatal conductance and relative growth rate in durum wheat. This correlation indicates that a plant that can hold its stomata opening has good salt tolerance. Nevertheless, under salt stress, limited stomatal opening can prevent water loss and wilting (Li et al., 2017; Hedrich and Shabala, 2018). Stomatal closure in the early periods of salinity stress has the same function as that in drought conditions (Munns, 2011).

Recently, the mechanism of abscisic acid (ABA) perception and signal transduction has been well studied in *Arabidopsis thaliana* (Lucas et al., 2013; Osakabe et al., 2014; Mittler and Blumwald, 2015). ABA is a prominent chemical signal that controls stomatal movement during the early period of stress (Sah et al., 2016). Under salinity conditions, Cl^−^ and Na^+^ induce the increase in leaf tissue ABA concentrations in plants exposed to 50 mM of these ions for 2 h; this result suggests that the early accumulation of ABA is useful in maintaining cell turgor (Geilfus et al., 2018). However, arguments related to ABA transport from the roots to the shoots and the regulation of stomatal movement still exists (Grondin et al., 2015; Cai et al., 2017; Li et al., 2018). Some reports support the view that root-sourced ABA leads to stomatal closure in the shoots (Davies et al., 2005; Jiang and Hartung, 2008). However, that root-to-shoot ABA transport is not the only way to induce stomatal responses. Grafting experiments have been conducted to determine the source of ABA in drought-induced stomatal closure. Some of these experiments proposed that leaf-sourced ABA is also necessary for stomatal closure under stress conditions (Holbrook et al., 2002). Apart from ABA concentration, ABA sensitivity in response to water deficit or salinity in the leaves affects stomatal movement (Wilkinson and Davies, 2002; Liu et al., 2015). Li et al. (2011) found that stomatal sensitivity to a root-sourced ABA signal gradually increased from the base to the apex along the vine of grapevine. Interestingly, the sensitivity of stomatal movement to ABA are affected by the type of roots. Cucumber with luffa as rootstock is more sensitive to ABA than cucumber as rootstock (Liu et al., 2015). Thus, in addition to ABA biosynthesis, the increased sensitivity of stomata to ABA might play an important role in rootstock-induced salt tolerance.

Our previous research showed that grafted cucumber onto pumpkin rootstock improved the salt tolerance of cucumber (Liu et al., 2012b; Lei et al., 2014; Niu et al., 2017). In this process, the limitation of Na^+^ uptake and transport from the roots to the shoots play critical roles. Most of the results regarding Na^+^ restriction by rootstock were obtained after prolonged salt treatments (from days to weeks). However, a recent report indicated an earlier rootstock-sourced signal (within several hours) triggered stomatal closure and contributed to enhanced salt tolerance by reducing water loss (Niu et al., 2018). Nevertheless, the mechanism behind this quick stomatal closure remains unknown.

In the present study, we evaluated photosynthetic parameters and ABA level in two grafting combinations (self-grafted cucumber and cucumber grafted onto pumpkin rootstock). Through ABA biosynthesis inhibitor experiments, we showed that root sourced ABA production confers salt tolerance on grafted cucumber plants by triggering stomatal closure within the first few hours of salt stress and optimizing plant ionic and water balance.

## Materials and Methods

### Grafting With Different Rootstocks

The experiment was carried out in a growth chamber at Huazhong Agricultural University, Central China. A salt-sensitive cucumber (*Cucumis sativus* L.) cv. Jinchun No. 2 (abbreviated as “C”) was used either as a scion or rootstock, and a salt-tolerant pumpkin (*Cucurbita moschata* Duch.) cv. Chaojiquanwang (abbreviated as “P”) as a rootstock. Two grafting combinations were used in this study: self-grafted cucumber plants (C/C) and pumpkin-grafted cucumber plants (C/P).

Seeds were soaked in distilled water for 6 h and incubated in the dark at 30°C until germination. The rootstocks were sown 4 days earlier than the cucumber scion. When rootstock seedlings developed one true leaf, the cucumber seedlings were grafted onto them by using the “hole insertion grafting” method described by Lee et al. (2010). After 7 days, the grafted plants were transferred to plastic containers (six seedlings per container) containing 8 L of full-strength Hoagland’s solution. The nutrient solution was replaced every 3 days and continuously aerated. At the four-leaf stage, the grafted plants were used for subsequent experiments.

### Salt Treatment and ABA Biosynthesis Inhibitor Application

The process of stomatal closure in the two grafting combinations were investigated. NaCl was added in the nutrient solution until a final concentration of 75 mM was obtained. Photosynthesis parameters were monitored 0, 0.5, 1, 3, 6, 24, 48, and 120 h after the commencement of the salt treatment. ABA content in the leaves and roots was measured.

In the inhibitor experiments, ABA inhibitor fluridon (Flu) (CAS:59756-60-4, Sigma Aldrich) at a concentration of 50 µM was sprayed on the leaves (5 ml per plant), whereas roots of others were submerged with 50 µM fluridon (Flu) for 6 h and then transferred to Hoagland’s solution containing 75 mM NaCl. An equal amount of water was applied as the control.

After 1 and 3 h, the photosynthetic parameters and ABA content were determined, respectively.

### Determination of Photosynthesis Parameters

Photosynthetic rate (Pn), transpiration rate (Tr), intercellular CO_2_ concentration (Ci), and stomatal conductance (Gs) were detected from the second recently expanded leaf with an open gas exchange system (Li-6400, Li-Cor, Inc., Lincoln, NE, USA). The assimilatory chamber was controlled to maintain the leaf temperature at 28°C, CO_2_ concentration at 360 µmol mol^-1^, and photosynthetic photon-flux density at 600 µmol m^-2^ s^-1^. Five replicates per treatment were measured between 08:30 and 11:30 AM.

### Xylem Sap Collection

Xylem sap was collected according to the method described by Liu et al. (2015) with some modifications. Briefly, stems were cut 2 cm above the grafting junction between the scion and rootstock with a razor blade, and then the rootstock was exposed to the pressure chamber set to 0.5 MPa. Contamination from injured cells at the cut surface was prevented by discarding the first two droplets of the xylem sap. Then, xylem sap was collected for 20 min with a micropipette from the cutting surface into ice-chilled Eppendorf tubes. The collected sap samples were frozen immediately in liquid nitrogen and stored at −80°C until analysis.

### Determination of ABA Content in the Leaves, Roots, and Xylem Sap

The samples were ground to a fine power in liquid nitrogen with a mortar and pestle then mixed with 750 µl cold extraction buffer (methanol: water: acetic acid, 80:19:1, v/v/v) supplemented with internal standards (10 ng ^2^H_6_ABA) (Olchemin). The samples were placed on a shaker for 16 h at 4°C in the dark, and then centrifuged at 10,000 rpm for 15 min at 4°C. The supernatants were combined and filtered by using a nylon filter (Nylon 66; Jinteng Co., Ltd, Tianjing, China). The filtrate was dried by evaporation under nitrogen gas flow at room temperature, and then dissolved in 200 µl of methanol. Liquid chromatography was carried out with an ultra-fast liquid chromatography-electrospray ionization tandem mass spectrometry (UFLC-ESI-MS) with an auto sampler (Shimadzu Corporation, Kyoto, Japan) as reported by Liu et al. (Liu et al., 2012a).

#### Stomatal Aba Sensitivity Experiment

The effect of rootstock on the stomatal sensitivity to ABA in scion leaves was investigated by observing stomatal movements with the second recently expanded leaf from the top of the two grafted combinations. The stomatal movements were measured by the method described by Liu et al. (2015) with some modifications. Briefly, epidermal strips were peeled from the abaxial surface with forceps, and mesophyll cells were removed from the epidermis with a single-edge industrial razor blade. The epidermal strips were submerged into stomatal opening buffer (10 mM KCl, 7.5 mM iminodiacetic acid, and 10 mM MES, pH 6.2, adjusted with KOH) for 2 h under fluorescent light (100 µmol m^−2^ s^−1^) and then incubated for 3 h in stomatal opening buffer supplemented with 0, 10, 20, 50, or 100 µM ABA before the stomatal openings were observed.

### Total RNA Extraction and Gene Expression Analysis

Total RNA was isolated from the roots with a TransZol reagent (TransGen Biotech, Inc., Beijing, China) according to the manufacturer’s instructions. After extraction, the total RNA was dissolved in the diethylpyrocarbonate-treated water. The cDNA template for the quantitative real-time PCR (qRT-PCR) was synthesized from 1 µg of total RNA with HiScript II Select RT SuperMix for qPCR (Vazyme, Piscataway, NJ, USA).

For qRT-PCR analysis, we amplified the PCR products in triplicate by using 1×Top Green qPCR SuperMix (TransGen Biotech, Inc., Beijing, China) in 10 µl qRT-PCR assays. The PCR was performed with ABI Prism^®^ 7000 (Applied Biosystems, USA), and the cycling conditions were as follows: denaturation at 94°C for 30 s, followed by 40 cycles of denaturation at 95°C for 5 s, annealing at 55°C for 15 s, and extension at 72°C for 15 s. The specific primers (**Table S1**) were designed based on the published mRNA of *Cucurbita moschata* on Cucurbit Genomics Database (http://cucurbitgenomics.org) by using Primer 5 software. The relative gene expression was calculated as previously described by Livak and Schmittgen (2001).

## Results

### Cucumber Grafted Onto Pumpkin Showed More Rapid Stomatal Closure than Self-Grafted Cucumber Under Salinity Stress

**Figure 1:**
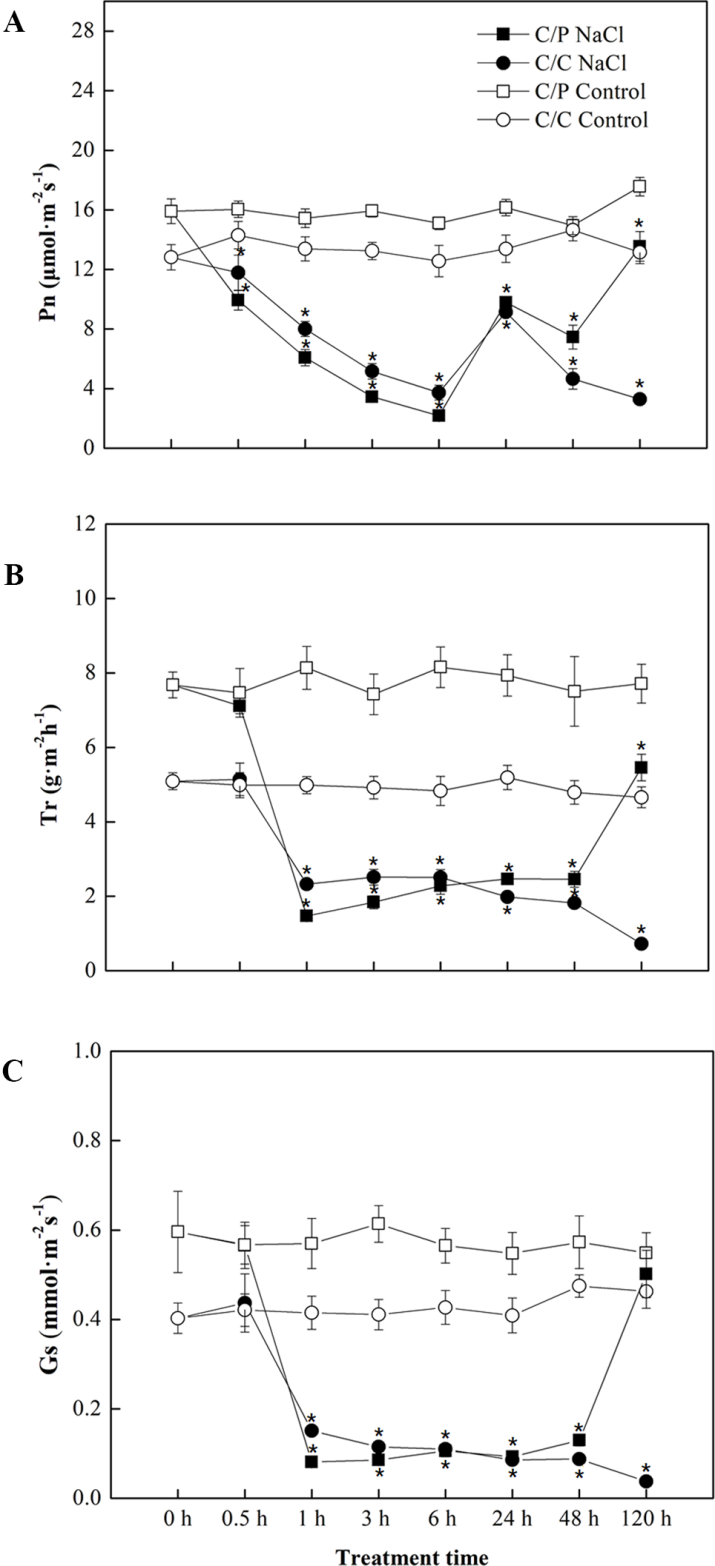
Time course of photosynthetic rate **(A)**, transpiration rate **(B)** and stomatal conductance **(C)** in the leaves of pumpkin-grafted cucumber (C/P) and self-grafted cucumber (C/C) plants after 75 mM NaCl treatment. Data are presented as the mean of six biological replicates ( ± SE), points with an asterisk indicate a significant difference from the untreated control (*P* 0.05) at the same sampling time according to *t*-test.

Salt treatment apparently reduced the Pn of pumpkin grafted and self-grafted cucumber plants by 14% and 25%, respectively, after 6 h of treatment (**Figure 1A**). However, Pn was recovered by up to 70% compared with the controls after prolonged treatment (24 h) in both grafting combinations. After 120 h of salt treatment, the Pn of C/P was 4.1 times of that of C/C (C/C almost died). Consistent with Pn, Tr and Gs showed a similar decline in the early period of salt treatment, but their responses were faster than the response of Pn. Minimum Tr and Gs were attained after 1 h of salt treatment. Interestingly, the C/P presented more rapid stomatal closure than self-grafted cucumber plants under salinity during the first 1 h of salt treatment (**Figures 1B, C**).

### Higher Concentration of ABA Was Detected in the Leaves Compared With Roots

**Figure 2:**
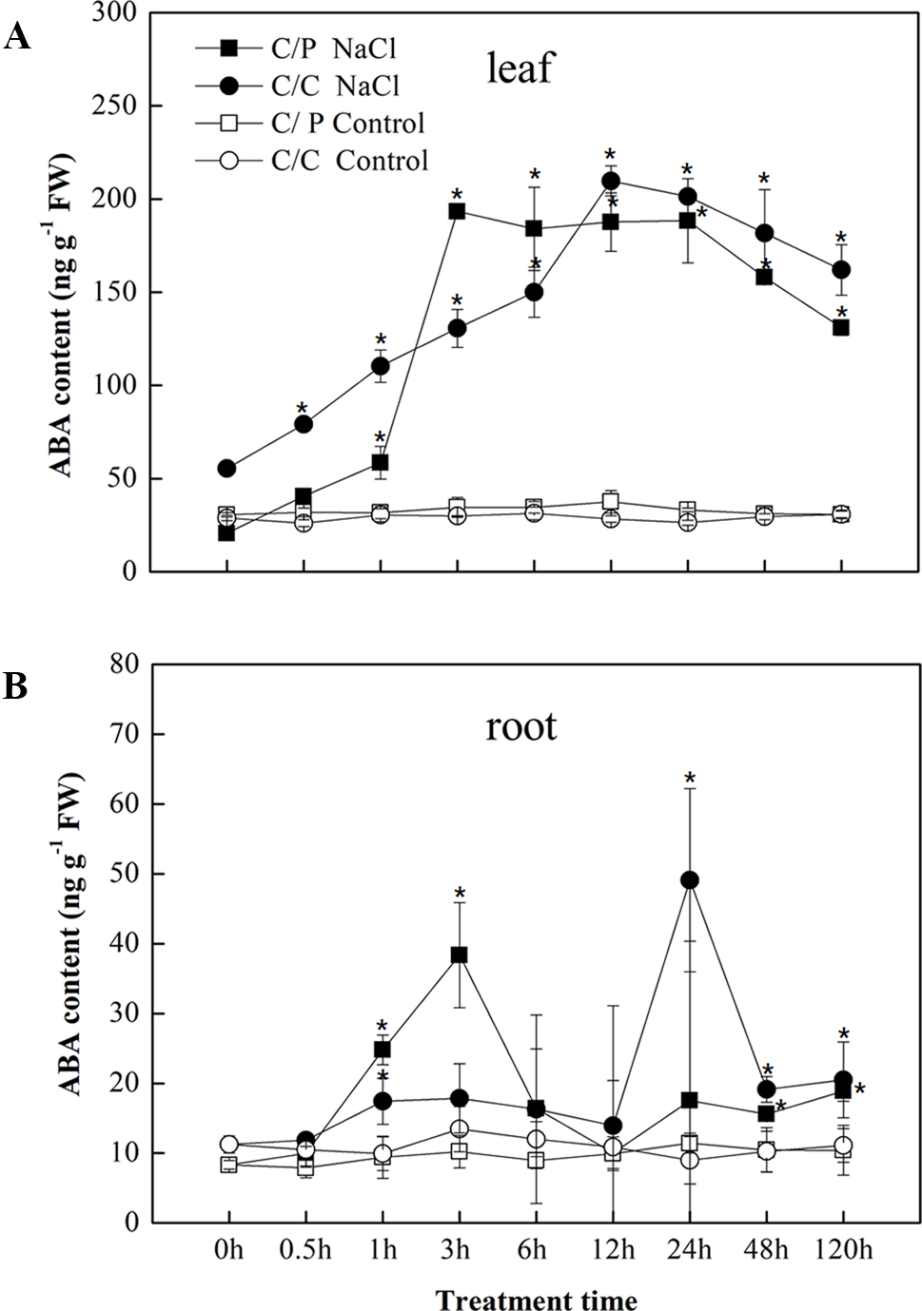
Abscisic acid (ABA) accumulation in the leaves **(A)** and root **(B)** of the pumpkin-grafted cucumber (C/P) and self-grafted cucumber (C/C) after 75 mM NaCl treatment (NaCl) or non-salinity condition (Control). Data are presented as the mean of three biological replicates. ( ± SE), points with an asterisk indicate a significant difference from the untreated control (*P* 0.05) at the same sampling time according to *t*-test.

In response to salt stress, ABA accumulation was observed in the leaves and roots of the C/P and C/C plants after salt treatment (**Figures 2A, B**). An apparent increase in ABA accumulation in the leaves was detected 1 h after exposure to salinity conditions and peaked at 3 h in C/P and 12 h in C/C. Compared with ABA contents in the leaves, the ABA contents in the roots sharply increased at 3 h in C/P and 24 h in C/C. The seedlings showed a higher ABA levels in the leaves than the roots throughout the observation period (120 h).

### Inhibition of ABA Biosynthesis in the Roots Compromises Salinity-Induced Rapid Stomatal Closure in Pumpkin-Grafted Cucumber

**Figure 3:**
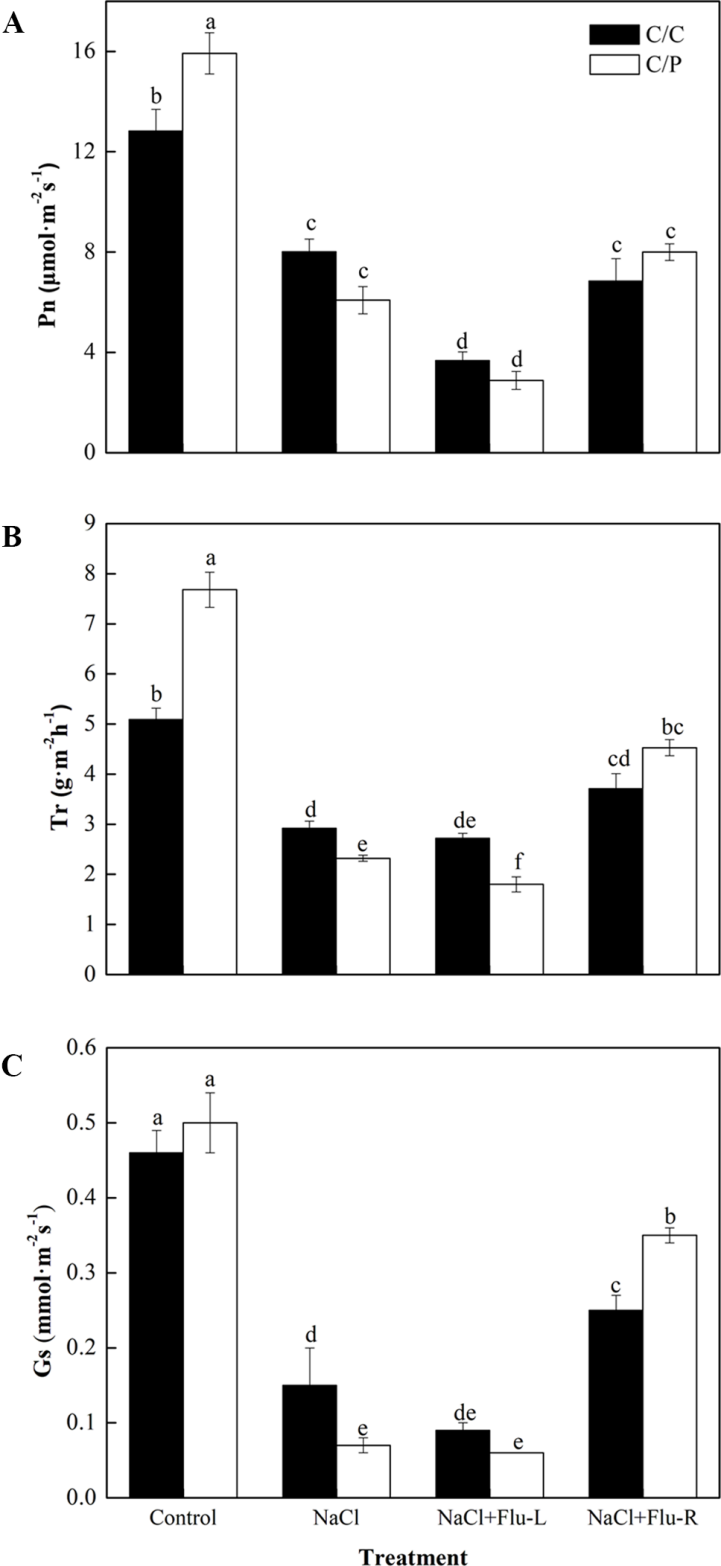
Effect of abscisic acid (ABA) synthesis inhibitor Flu on photosynthetic rate **(A)**, transpiration rate **(B)** and stomatal conductance **(C)** of pumpkin-grafted cucumber (C/P) and self-grafted cucumber (C/C) after 1 h of NaCl treatment. The seedlings were pretreated with Flu on leaves (NaCl+Flu-L) or on roots (NaCl+Flu-R), respectively. Data are presented as the mean of six biological replicates ( ± SE). Different letters indicate significant differences (*P* 0.05) according to the Duncan’s multiple test.

Flu is an efficient inhibitor that blocks the biosynthesis of ABA in plants (Hossain et al., 2011; Li et al., 2019). To study the relationship between ABA synthesis and stomatal movement, we determined the changes in photosynthetic parameters in C/C and C/P plants with foliar or root application of Flu. The results showed that the response for stomatal movement was tissue specific. Leaves pretreated with Flu decreased the Pn of the C/C and C/P plants but only reduced the Tr in the C/P plants. By contrast, root pretreatment with Flu showed a pronounced reverse effect on Tr and Gs, and the Gs in Flu-treated plants was 5.5 times of that in non-Flu-treated C/P plants exposed to salinity stress (**Figures 3B, C**). In other words, the stomatal closure repressed by ABA inhibitor treatment was more extensive in the roots than in the leaves.

### ABA Inhibitor Treatment with Root Caused a Reduced Level of ABA in Roots, Xylem Sap, and Leaves in Pumpkin-Grafted Cucumber

**Figure 4:**
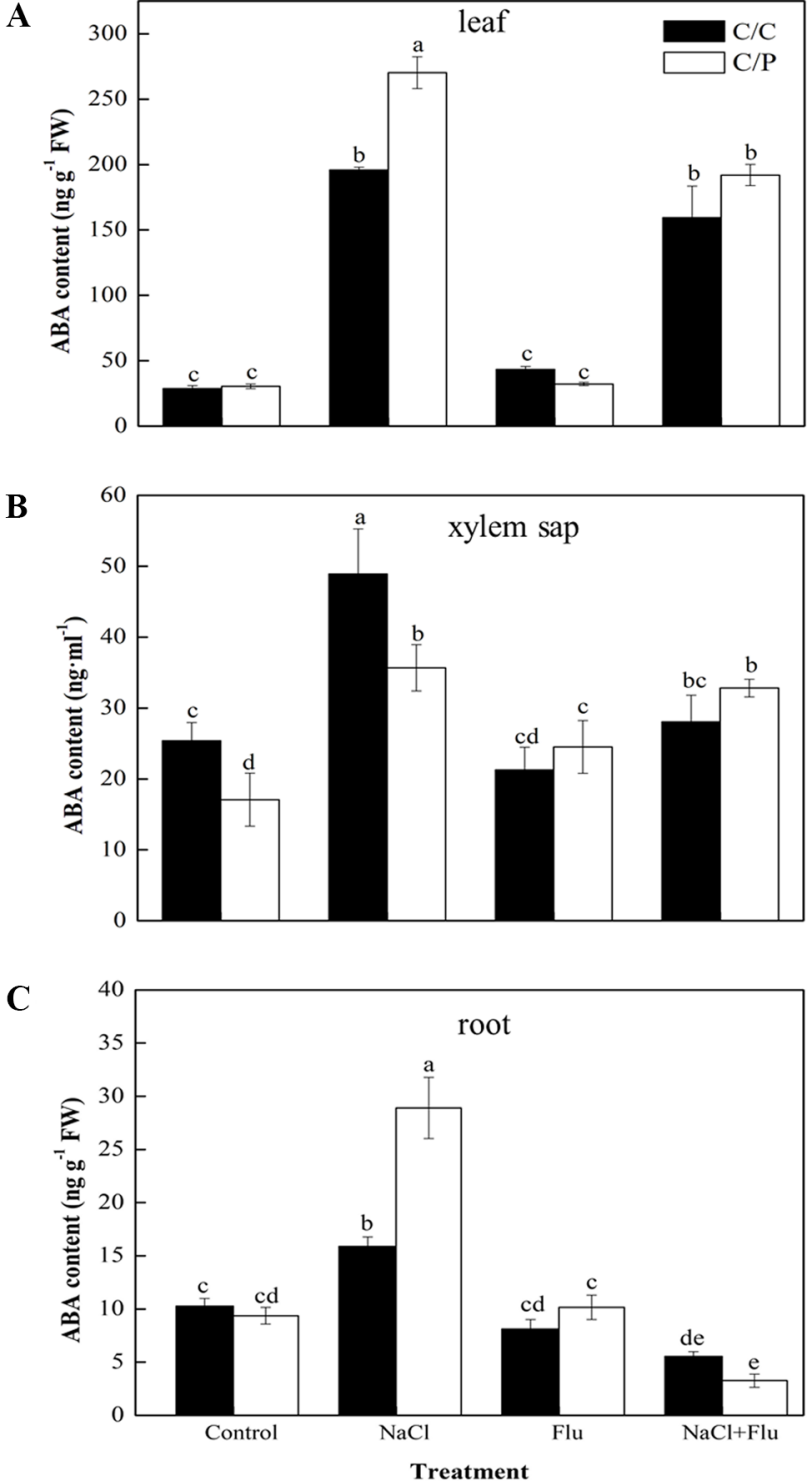
Effect of abscisic acid (ABA) synthesis inhibitor Flu on ABA contents of the leaves **(A)**, xylem sap **(B)**, and roots **(C)** of the pumpkin-grafted cucumber (C/P) and self-grafted cucumber (C/C) plants after 3 h of NaCl treatment. Data are presented as the mean of three biological replicates ( ± SE). Different letters indicate significant differences (*P* 0.05) according to the Duncan’s multiple test.

To study the mechanism by which rootstock ABA levels affect stomatal movement in the scion, we measured the ABA levels in the roots, xylem sap, and leaves with or without Flu-pretreatment. NaCl treatment obviously increased the ABA content of the roots, xylem sap, and leaves (**Figures 4A–C**). These results were consistent with the ABA trends shown in **Figure 2**. The ABA levels in the xylem sap under NaCl treatment were 1.93 and 2.93 times of those in the control plants of C/C and C/P, respectively (**Figure 4B**). Flu-treatment alone had no significant effect on ABA levels in the leaves, but the ABA content in the xylem sap of C/P slightly increased compared with that of non-Flue-treated C/P plants (**Figure 4B**). Flu-pretreatment significantly reduced the ABA levels in the leaves, roots, and xylem sap of C/C and C/P under salinity stress conditions. In the three plant samples, Flu had the strongest inhibitory effect on roots that presented 34.70% and 11.30% ABA concentrations compared with the ABA concentrations of non-inhibitor-treated C/C and C/P plants, respectively.

### Increased ABA Sensitivity Was Observed for Pumpkin Grafted Cucumber Plants

**Figure 5:**
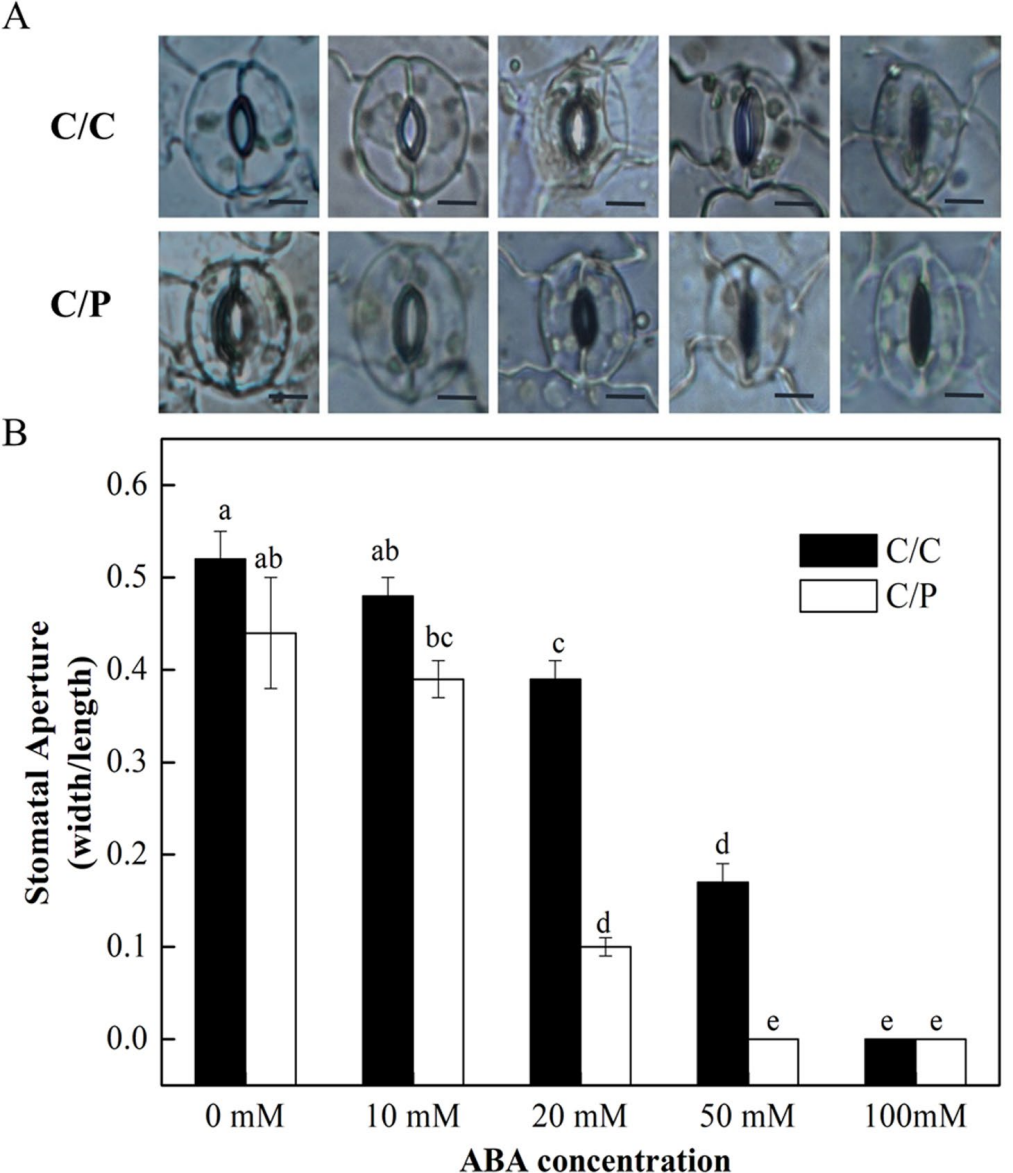
The effect of abscisic acid (ABA) concentration on stomatal opening in detached abaxial epidermal strips of pumpkin-grafted cucumber (C/P) and self-grafted cucumber (C/C) plants. The fields are provided by a optical microscope (OLYMPUS CX31) under 100× magnification **(A)** and stomatal aperture are calculated with width/length **(B)**. Data are presented as the mean of six biological replicates ( ± SE). Different letters indicate significant differences (*P* 0.05) according to the Duncan’s multiple test. Scale bar in **Figure 5A** = 10 μm.

The rootstock-promoted stomatal closure depends on an increased sensitivity to ABA under salinity. The detached leaves from C/C and C/P were used in the measurement of stomatal movement under different concentrations of exogenously applied ABA. The results showed that the stomatal opening was decreased with increasing ABA concentration (**Figure 5B**). Notably, ABA at the concentration of 20 µM apparently induced stomatal closure in C/C and C/P plants. However, the aperture value was 0.39 (width/length) in C/C and only 0.1 in C/P plants (**Figure 5B**). These results suggested that stomata in C/P plants had an increased sensitivity to ABA compared with C/C plants.

### ABA Synthesis Related Genes Expression Pattern

**Figure 6:**
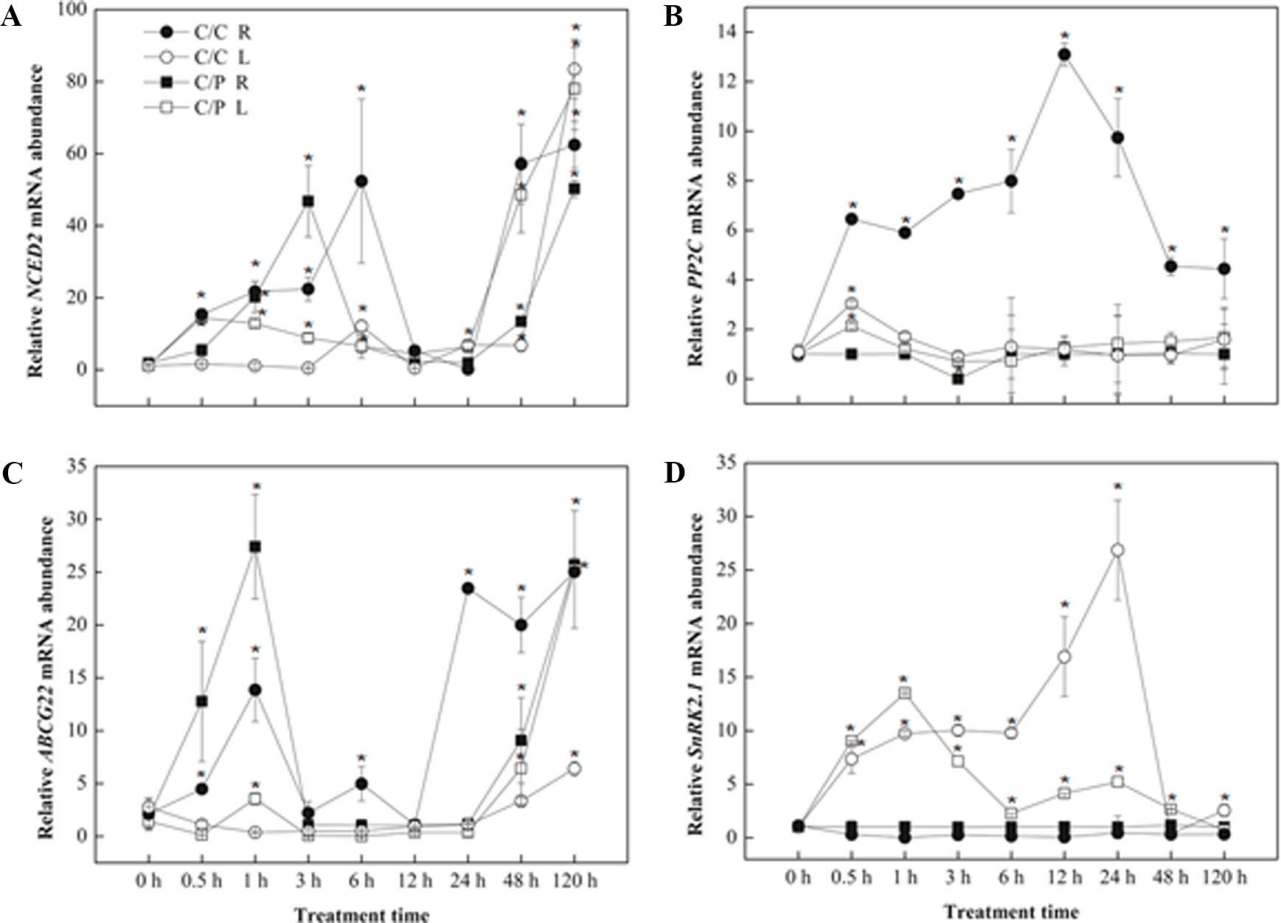
Transcript abundance of abscisic acid (ABA) biosynthesis related genes [NCED2 **(A)**,PP2C **(B)**,ABCG22 **(C)** and SnRK2,1 **(D)**] in the leaves (L) and roots (R) of pumpkin-grafted cucumber (C/P) and self-grafted cucumber (C/C) plants. Data are presented as the mean of three biological replicates ( ± SE), points with an asterisk indicate a significant difference from the untreated control (*P* 0.05) at 0 h according to *t*-test.

The relative expression levels of four genes related to ABA synthesis or signaling pathway were measured by qRT-PCR (**Figures 6A–D**). *NCED2* and *ABCG22* have similar response pattern in both grafting combinations. An increased expression of these two genes was observed during the first several hours (6 h with *NCED2* and 1 h with *ABCG22*) in the roots. However, the expression of both genes decreased after reaching the peak and finally recovered again after 24 h. *NCED2* and *ABCG22* did not show increased expression in the leaves and showed increased expression only after prolonged salt treatment. The difference between the expression patterns of *NCED2*, *ABCG22*, and *SnRK2,1* was induced by salt stress only in the leaves, and the peak in C/C lagged behind compared with C/P.

## Discussion

### Rapid Stomatal Closure Might Be an Alternative Strategy to Overcome Salt Stress

Grafting cucumber onto resistant rootstocks improves plant tolerance to salinity; however, enhanced tolerance in grafted seedlings has been attributed to Na^+^ restriction (Lei et al., 2014). Herein, we found that cucumber plants grafted onto pumpkin had more rapid stomatal closure than self-grafted cucumber plants. This mechanism may contribute to the enhanced adaptability of plants to early osmotic stress under salinity condition (Shu et al., 2016; Li et al., 2018).

Stomata respond to rapid changes in the water potential of a soil solution. Stomatal conductance can be used as a screening trait for osmotic stress tolerance when stomatal response is induced by osmotic pressure outside the roots rather than by Na^+^ accumulation in the leaves. Photosynthesis was reduced first by the stomatal limitations, and nonstomatal effect was not observed in the first few hours or days after salt treatment or until Na^+^ or Cl^−^ accumulated in leaves by up to nearly 250 mM (James et al., 2006). In our previous work, we observed that the seedlings did not accumulate Na^+^ to toxic levels (not more than 5 mgg^−1^, DW) until 24 h (Niu et al., 2018). The present study showed that the seedlings close their stomata within the first hour after salt treatment (**Figure 1**). This result shows that quick stomatal response is induced by osmotic stress rather than Na^+^ accumulation. Previous studies showed that rapid reduction in stomatal aperture and transpiration in response to salinity is not due to the osmotic pressure of the salt alone because nonionic osmoticum have a similar effect (Tardieu et al., 2015). According to our previous study, we observed that NaCl and isosmotic sorbitol induce rapid stomatal closure during the first 4 h of salt treatment (Niu et al., 2018).

Hydraulic signals under salinity stress may dominate and trigger the response of stomata in the leaves and plants under water stress (Christmann et al., 2007). Until now, our knowledge regarding the mechanism of grafting-induced early period salinity tolerance is incomplete. In this study, pumpkin grafted cucumber plants had a quick stomatal response under salinity conditions compared with self-grafted cucumber plants. Cucumber grafted onto pumpkin have an increased ability to sense salinity stress compared with self-grafted cucumber plants.

### Root or Leaf, Who Is the Key Position to Induce the Stomata Closure?

The enhanced ABA level in the leaves and xylem sap of the two grafting combinations suggests that cucumber and pumpkin might have the similar osmotic stress response mechanism (**Figure 4**). Several works related to ABA and stomatal movement were performed, but whether ABA transported from the roots to the shoots regulates stomatal movement remains disputable (Davies et al., 2005; Schachtman and Goodger, 2008; Wilkinson and Davies, 2010). In this study, the two grafting combinations shared a similar ABA responding trend, and remarkable increase of ABA was detected in leaves compared with roots (**Figure 2**). These results indicated that ABA concentration at the action site (leaf) should be considered as the real ABA signal that promotes stomatal closure. Similar results were found in maize; the ABA concentration significantly increased in the leaves of salt-resistant maize SR03; this change was not observed in salt-sensitive hybrid lector (Zörb et al., 2013). However, ABA inhibitor experiments showed that the Flu-pretreatment of roots lead to more acute limited stomatal closure than the Flu-pretreatment of leaves (**Figures 3A–C**). This finding indicated that the root-synthesized ABA could be an initial signal triggers the stomatal closure action in leaves. ABA-related genes expression in the roots and leaves provides some clues (**Figure 6**). Most genes involved in ABA biosynthesis were activated in the roots during the first 12 h of salt treatment. By contrast, except *SnRK2,1*, no gene was activated in the leaves. We speculated that the long-distance ABA signal motivated the stomata action in leaves. ABA accumulation in the roots induced by salt stress and triggered by osmotic stress leads to ABA accumulation in the leaves. Similar results were found in citrus. The tetraploid citrus clones show an increased tolerance to drought compared with the diploid ones by up-regulating the expression of genes involved in long-distance ABA signaling in the roots (Allario et al., 2013). A significantly reduced ABA in the xylem sap was observed after the roots were subjected to Flu pretreatment (**Figure 4**). This observation was consistent with previous study on *Arabidopsis*, in which stomatal responses were correlated with ABA content in the xylem rather than the ABA content in the leaf, suggesting the importance of ABA as a long-distance signaling hormone in the xylem (Jiang and Hartung, 2008). Notably, Flu pretreatment in the roots cannot completely inhibit increase in ABA level under salinity condition. Thus, the other parts of plants may have the same ability to synthesize ABA. Vascular tissues are important to ABA biosynthesis under dehydration stress.

### Mechanism of Increased Stomatal Sensitivity to ABA in Pumpkin Grafted Cucumber Plants

We presume that the reason that makes C/P plants to close stomata earlier than the C/C plants is the enhanced responsiveness of the scion to ABA. This hypersensitivity to ABA is related to enhanced tolerance to salt through the regulation of stomatal closure and expression of stress-related genes. Plants exposed to osmotic stress utilize the ABA signaling transduction pathway to initiate the expression of defense genes (Kumar et al., 2017). The overexpression of some stress-related genes, such as *OsZIP72* and *OsABI5*, results in abiotic-stress tolerance and causes transgenic plants to be hypersensitive to exogenous ABA (Lu et al., 2009). In our study, stomatal movement in response to salinity in the leaves of C/P plants was associated with increased sensitivity to ABA, as evidenced by the decreased rate of concentration-mediated stomatal closure (**Figure 5**). ABA sensing and signaling are mediated by several key proteins, such as PP2C and SnRK2 (Yoshida et al., 2015). According to a previous study, the improved ABA sensitivity of stomata in luffa grafted cucumber is associated with the differential expression of these two genes (Liu et al., 2015). SnRK2 interacts with several proteins, including kinases, phosphatases, transcription factors, and metabolic enzymes, and stomatal opening mediated by KAT1 might be negatively regulated by phosphorylation by SnRK2 (Yoshida et al., 2015). During this process, a slow anion channel (SLAC1) may play important role (Jones et al., 2014).

However, how these genes alter the sensitivity of grafted plants to ABA requires further investigation.

## Conclusion

We demonstrate that pumpkin rootstock obviously improves the osmotic stress tolerance of cucumber scion under salinity stress conditions. In this process, rootstock-sourced ABA serves as a key signal to mediate rapid stomatal closure in the cucumber scion, thus preventing wilting in the early period of salinity stress. We also observed that the plants of both grafting combinations shared a similar ABA synthesis and response pattern. The increased sensitivity of stomata to ABA in the leaves plays an important role in the action of rapid stomatal closure. Alterations in the expression of some key genes involved in ABA biosynthesis, perception, signaling, and transport were found to be associated with rootstock-mediated salt tolerance. This quick responsive mechanism can be useful in improving agricultural production particularly in salt affected areas.

## Data Availability Statement

All datasets generated for this study are included in the article/**Supplementary Material**.

## Author Contributions

ZB and MLN designed research. MN, SS, JS, JL, and HC performed research. YH analyzed data. MLN and MN wrote the paper.

## Funding

This work was supported by the National Key Research and Development Program (2018YFD100800), the National Natural Science Foundation of China (31572168, 31772357), and the Hubei Provincial Natural Science Foundation of China(2019CFA017).

## Conflict of Interest

The authors declare that the research was conducted in the absence of any commercial or financial relationships that could be construed as a potential conflict of interest.
