# Supplementary material for: Grafting Cucumber Onto Pumpkin Induced Early Stomatal Closure by Increasing ABA Sensitivity Under Salinity Conditions
Source: Front Plant Sci. 2019 Nov 11;10:1290. doi: 10.3389/fpls.2019.01290 (PMC6859870; doi:10.3389/fpls.2019.01290)
Supplement: Supplementary file 1 [file Table_1.docx]

**Supplementary Table 1**

The list of primer sequences used for qRT-PCR analysis.

| Grafted combinations | Genes | Forward Primer | Reversed Primer | Gene ID |
| --- | --- | --- | --- | --- |
|  |  |  |  |  |
| C/C | *NCED2* | GTGAACCGAAATCTACTTG | GCTCACCACCATACCTC | CsaV3_6G051790 |
|  | *SnRK2.1* | TCGCAACCTTCTTTCTCGC | ATTTCCTCAACGCTCTGTG | CsaV3_2G016930 |
|  | *ABCG22* | GGAAGGGAATAATACGACAG | TCTGCTCCCAACATAAACAT | CsaV3_4G007160 |
|  | *PP2C1* | TTATGGAGACTGATGCAGCT | ATCTCCTATGGCACGTGTAAC | CsaV3_3G011120 |
|  | *EF1a* | ACTGTGCTGTCCTCATTATTG | AGGGTGAAAGCAAGAAGAGC | CsaV3_4G033290 |
| C/P | *NCED2* | AGGGTGAAAGCAAGAAGA | AGGGTGAAAGCAAGAAGAGC | CmoCh16G005470 |
|  | *SnRK2.1* | GGCATACATAGCACCAGA | AAGCACCAACCAACATAAC | CmoCh01G020750 |
|  | *ABCG22* | GGAAGTCCAGGAGTGCA | GAAGGAGAATGACAAACCC | CmoCh18G009810 |
|  | *PP2C1* | CGGTTCCTCTTGTTTGG | ATTTCACATTCACTTCCCT | CmoCh11G004060 |
|  | *EF1a* | GGCTCCTTCTCGAGTTCCTT | GGAAGTCCAGGAGTGCA | CmoCh17G001050 |

All primers were designed based on a published mRNA on Cucurbit Genomics Database (http://cucurbitgenomics.org) using Primer 5 software. *EF1*a is the reference gene.
